# Molecular Dynamics Study of Hydrogen Release from NaH Using Machine Learning Potential

**DOI:** 10.3390/ma19153308

**Published:** 2026-08-04

**Authors:** Ce Feng, Yuting Zhang, Xiao Zhang, Shikai Chang, Fuhao Zhang, Linyuan Cao, Jingya Dong, Rongdong Wang

**Affiliations:** 1 Department of Reactor Engineering Technology, China Institute of Atomic Energy, Beijing 102413, China; fengce@cnncmail.cn (C.F.); zhangyuting@cnncmail.cn (Y.Z.); dgs_zhangxiao@cnncmail.cn (X.Z.); zhangfuhao@cnncmail.cn (F.Z.); 2 School of Chemical Engineering and Technology, Tianjin University, Tianjin 300350, China; 3 Jiangsu Key Laboratory for Carbon-Based Functional Materials & Devices, Institute of Functional Nano & Soft Materials (FUNSOM), Soochow University, Suzhou 215123, China; 20244014009@stu.suda.edu.cn; 4 Institute of Nuclear and New Energy Technology, Tsinghua University, Beijing 100084, China

**Keywords:** sodium hydride, decomposition behavior, sodium-cooled fast reactor, deep neural network potential, DP-GEN active learning framework

## Abstract

Sodium hydride (NaH) is the main by-product generated during the operation of the cold traps in a sodium-cooled fast reactor. Its thermal decomposition releases hydrogen gas, posing a safety hazard. However, understanding the atomic-scale decomposition mechanism remains challenging because conventional simulation methods are limited in the accessible length and time scales. This study developed a deep neural network potential (DP) for the NaH system using the DP-GEN active learning framework. Benchmark tests show that the DP model accurately reproduces density functional theory (DFT) reference energies, forces, equations of state, elastic properties, and phonon spectra. In particular, the DP-predicted bulk modulus and lattice constant are in good agreement with DFT results and close to experimental values, significantly outperforming the empirical ReaxFF potential. Subsequently, we conducted large-scale Deep Potential Molecular Dynamics (DPMD) simulations to investigate the thermal decomposition behavior of NaH clusters and a slab model. The simulation results reveal model-dependent thermal responses of NaH. In the original Na_48_H_48_ cluster simulation heated from 100 to 1200 K, a structural transition and disordering were observed, but no H_2_ formation occurred within the simulation time. In contrast, the slab model heated from 300 to 1500 K exhibited surface disordering, Na-H bond cleavage, H-H bond formation, cluster detachment, and H_2_ formation and release at elevated temperatures. A supplementary higher-temperature Na_48_H_48_ cluster simulation further showed cluster dissociation in the 1000–1500 K range. These results provide atomistic insight into the model- and temperature-dependent decomposition behavior of NaH and suggest that the DP model is a useful tool for studying hydrogen-related processes in alkali metal hydrides.

## 1. Introduction

A cold trap is a crucial purification facility for the primary and secondary coolant loops of sodium-cooled fast reactors (SFRs) [1]. During operation, hydrogen and oxygen impurities are trapped and deposited in the wire mesh mainly in the form of NaH and Na_2_O [2]. The continuously formed hydrogen and its isotope tritium pose risks to cold trap disposal [3]. Therefore, understanding the thermal decomposition behavior of sodium hydride is of great significance for the safe disposal or regeneration of cold traps.

Several studies have investigated the thermal decomposition behavior of NaH [4,5,6,7,8,9]. Murugesan et al. experimentally determined the temperature dependence of the NaH decomposition rate and hydrogen release rate and established the reaction order and activation energy as functions of the extent of decomposition (α) [10]. Kawaguchi’s work characterized the temperature dependence of the NaH thermal decomposition rate and reported an inflection point near 620 K, which was attributed to the presence of two types of hydrogen (fast-diffusing and immobilized) [11]. In addition, theoretical approaches such as density functional theory (DFT) have provided new tools and insights for studying NaH decomposition. Ojwang et al. developed a ReaxFF force field for NaH based on an ab initio training dataset and investigated the properties and thermal decomposition behavior of small NaxHy clusters, and obtained trends consistent with experimental observations [12]. Hao et al. used DFT to study hydrogen defects in NaH and indicated that appropriate defects can enhance hydrogen diffusivity [13]. Similarly, Singh et al. employed DFT to calculate vacancy-mediated hydrogen migration barriers in bulk NaH and near the NaH(001) surface, and used a hopping diffusion model to estimate hydrogen transport rates in agreement with experiments [14]. However, a key gap still remains in the above studies: NaH thermal decomposition and the associated hydrogen migration/defect evolution often take place on much larger spatiotemporal scales, requiring long-time dynamical sampling in complex environments such as nanograins, surfaces/interfaces, and defect-rich regions. DFT-level calculations are unable to sufficiently access large system sizes and time scales, while the accuracy and transferability of conventional empirical force fields (e.g., ReaxFF) across different phases, defect types, and interfacial environments still require further validation. Therefore, there is an urgent need for a potential that combines near-first-principles accuracy with the efficiency of molecular dynamics to systematically characterize the structure and dynamics of NaH.

In recent years, machine learning-assisted atomic simulation methods have achieved great success and received widespread attention [15]. Machine learning potentials (MLPs) are trained on reference electronic-structure data and can retain the accuracy of the reference method while enabling much more efficient molecular dynamics simulations. Furthermore, Bayerl et al. [16]. have pointed out that material property predictions based on MLPs converge faster and have less fluctuation in Brillouin zone integral accuracy than DFT-based predictions, thus making MLP far faster than DFT in calculating material properties. Deep neural networks are often used to learn the relationship between atomic configuration and the resulting energy, force, and stress. Importantly, once trained, these models can approach ab initio accuracy without explicitly solving the electronic-structure problem during molecular dynamics simulations. Among them, the Deep Potential Molecular Dynamics (DPMD) method [17,18,19] stands out as a promising approach and has been applied in the study of melting [20,21,22], phase transition [23], and phase behavior [24,25]. For example, the DPMD method has been applied to study the phase transition characteristics of alkali metal chloride salts [20], the phase diagram of ice [23], and the phase transition of carbon at high temperatures [24]. In summary, DPMD offers substantially higher efficiency and scalability than first-principles methods such as DFT, making it suitable for large-scale and long-time atomistic simulations. In this work, we trained a deep neural network potential for the NaH system using the DP-GEN active-learning framework. Benchmarking results show that the DP achieves accuracy comparable to first-principles calculations. The DP was then employed to investigate hydrogen dynamics in NaH clusters, elucidating the evolution of hydrogen content and the system energy. To gain deeper insight into structural transformations of NaH during thermal decomposition, we carried out heating runs in molecular dynamics simulations and observed the dissociation of NaH clusters at elevated temperatures.

## 2. Methods

### 2.1. DP Framework

In the DP framework, the potential-energy surface is represented by a neural network that maps local atomic environments to atomic energy contributions. In this system, the potential energy E is obtained by summing the energy $E^{i}$ of each type of atom in type i, that is

(1) $$E=\sum_{i}\;E^{i}$$

The local environment information of type i atoms within the cutoff radius $R_{c}$, denoted as $R_{c}^{i}$, is mapped by the embedding network to a feature matrix $D^{i}$. The embedding network preserves the rotational, translational, and permutation symmetries of $D^{i}$. Finally, $D^{i}$ is mapped to $E^{i}$ through a fitting network. Here, $R_{c}$ is set to 8 Å, with a smooth decay from 3 Å to 8 Å based on the inverse distance 1/r to eliminate discontinuities caused by truncation. The translational, rotational, and permutational symmetry are preserved by the descriptors $D^{i}$. The sizes of the embedding and fitting networks are (25, 50, 100) and (240, 240, 240), respectively. The residual network is employed in the training of the fitting network. The loss function is defined as:

(2) $$L(p_{\varepsilon },p_{f},p_{\xi })=p_{\varepsilon }\Delta \in^{2}+\frac{p_{f}}{3N}\sum_{i}\;|\Delta F_{i}{|}^{2}+\frac{p_{\xi }}{9}∥\Delta \xi {∥}^{2}$$

where Δε, $\Delta F_{i}$, and Δξ represent the root mean square errors of energy, force, and the virial tensor, respectively. $p_{\varepsilon }$, $p_{f}$, and $p_{\xi }$ are weight coefficients of energy, force, and the virial tensor. In our work, $p_{\varepsilon }$ increases from 0.02 to 2, $p_{\xi }$ increases from 0.01 to 1, and $p_{f}$ decreases from 1000 to 1. When the accuracy requirement is not met, the DP models are trained with 100,000 steps. After the accuracy criteria were satisfied, final training was performed for 1,000,000 steps.

### 2.2. DP-GEN Workflow

DP-GEN enables the automated construction of training datasets through an active-learning workflow consisting of model training, configuration exploration, and DFT labeling [23]. The initial training dataset is obtained from ab initio molecular dynamics (AIMD), and four DP models are trained with different initial deep neural network (DNN) parameter values. One of these DP models is then used for MD simulations to explore structural information under varying temperatures and pressures, while the other three DP models predict the energies and atomic forces for all structures in the MD trajectory. The maximum force deviation ($\sigma_{f}^{max}$) among the predictions of the four DP models serves as the criterion for labeling:

(3) $$\sigma_{f}^{max}=\underset{i}{max}\;\sqrt{\left\langle ∥F_{i}-\langle F_{i}\rangle {∥}^{2}\right\rangle }$$

where $\left\langle ∥F_{i}-\langle F_{i}\rangle {∥}^{2}\right\rangle$ denotes the average of the model predictions. The configurations with a small model deviation ($\sigma_{f}^{max}<\sigma_{low}$) are already well-predicted by four different DP models and are labeled as accurate configurations. Conversely, configurations exhibiting large model deviation ($\sigma_{f}^{max}>\sigma_{high}$) are highly distorted, unphysical, and insignificant, and are thus labeled as failed configurations. Those configurations satisfying $\sigma_{low}<\sigma_{f}^{max}<\sigma_{high}$ are labeled as candidates for further DFT calculations and are added to the training dataset for the next iteration. Here, $\sigma_{low}$ and $\sigma_{high}$ are set to 0.15 and 0.30 eV/Å, respectively.

### 2.3. DFT Calculation Setting

All DFT calculations are performed via the Vienna Ab initio Simulation Package (VASP 5.4.1) [26,27]. A generalized gradient approximation (GGA) in the form of the Perdew–Burke–Ernzerhof (PBE) functional is applied to account for the electronic exchange–correlation interaction [28]. The cut-off energy of the plane-wave basis set is 400 eV. The number of K-points is controlled by KSPACING and KGAMMA which are set to 0.40 Å^−1^ and True (Figure S1). The convergence tolerances of force and energy for geometric optimization are set as 0.03 eV/Å and 1 × 10^−6^ eV, respectively. AIMD simulations are performed in canonical (NVT) ensemble with a Nosé–Hoover thermostat and a 1 fs time step [29].

### 2.4. MD Simulation Settings

MD simulations are performed with the LAMMPS 2Aug2023 Software Package [30]. During the exploration step, MD simulations are performed under both isobaric–isothermal (NpT) and NVT ensemble. The temperature is controlled via a Nosé–Hoover thermostat, while the pressure is regulated using a Parrinello–Rahman barostat [29,31].

## 3. Results

### 3.1. Accuracy of the DP Model

**Figure 1:**
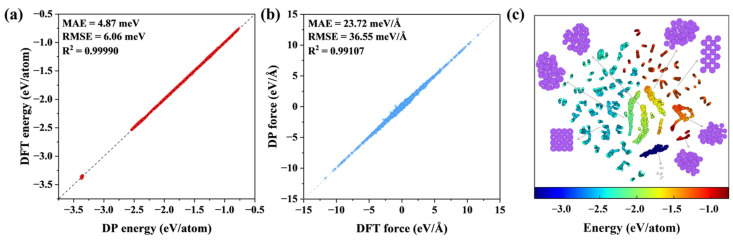
Comparison of DP-predicted and DFT-calculated (**a**) energies and (**b**) forces. (**c**) The 8414 atomic configurations in the training dataset were projected using a two-dimensional model after t-sne dimensionality reduction, where each dot corresponds to a configuration, and colors represent the energies of structures. The diversity of the data set is illustrated by representative atomic configurations from different regions.

The parity plots of energies and forces confirm the high accuracy of the DP model. (Figure 1a,b). For training data evaluation, the trained DP model yielded a root mean square error (RMSE) of 6.06 meV/atom for energies and 36.55 meV/Å for forces, with a corresponding mean absolute error (MAE) of 4.87 meV/atom and 23.72 meV/Å. The coefficients of determination (R^2^) of energies and forces are 0.9990 and 0.99107, respectively. The DP model also maintains good accuracy for configurations outside the most densely sampled region of the dataset. These results collectively validate that the trained DP model effectively demonstrates precise characterization of both energetic and mechanical properties within the system. Figure 1c exhibits the sampled configuration space of the training dataset, confirming its extensive coverage across a vast region of the structural space. These configurations were derived from the initial training structures including three-dimensional bulk structures (NaH and Na) and gaseous (H_2_) structures., as well as the cluster structures (Na_x_H_y_) generated using DP-GEN, as listed in Table 1. The final training dataset contains 8414 structures and 496,776 atoms in total. Table 2 provides a brief overview of these data, showing different types of structures.

**Figure 2:**
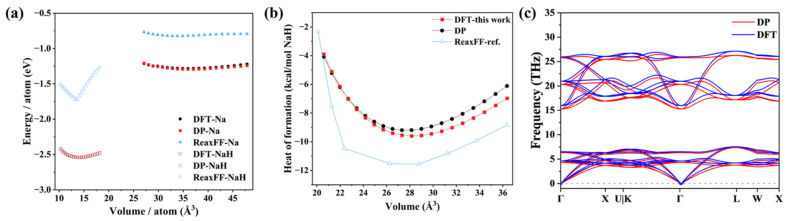
(**a**) Equation of state curves of NaH (*Fm3̅m*) and Na (*Im3̅m*) using DFT, DP model and ReaxFF [12]. (**b**) Heat of formation for NaH as computed from DFT, DP model and ReaxFF [12]. (**c**) Phonon dispersion relation of NaH (*Fm3̅m*) calculated by DP (red lines), and DFT calculations (blue lines).

To examine whether the DP model can accurately describe the expansion and compression behavior of NaH and Na crystals, we used DPMD to calculate the energy changes of NaH (*Fm3̅m*) and Na crystals (*Im3̅m*) under scaling conditions of 0.9~1.1, and compared the results with those of ReaxFF and DFT. Figure 2a shows that the DPMD calculation results are close to those of DFT, while the predicted average atomic energy of ReaxFF is much lower than that of DFT and DPMD, indicating a difference in energy baselines between the two models. From the perspective of crystal energy changes, ReaxFF accurately describes the energy changes of Na unit cells caused by scaling, but it performs poorly for NaH unit cells, deviating from the normal energy changes. Although the energy baselines of different models are different, the energy differences calculated by each model are comparable. Therefore, the heat of formation of NaH calculated by DPMD, ReaxFF, and DFT were compared, and the results are shown in Figure 2b. Within the scaling range of 0.9~1.1, the highest heat of formation of NaH calculated by ReaxFF is −11.8 kcal/mol NaH, which is 2.2 kcal/mol NaH higher than the result calculated by DFT. The DPMD results differ from the DFT results by only 0.4 kcal/mol NaH. This indicates that DPMD can accurately describe NaH and Na crystals. The DPMD results reproduce the DFT energy–volume relationship with high fidelity, indicating that the DP model accurately captures the underlying potential energy surface.

The structural properties of NaH are computed by doing a fit to the total energy versus cell-volume data, as calculated by DPMD, ReaxFF and DFT using the Birch–Murnaghan EOS [32]. As shown in Table 3, the bulk modulus ($B_{0}$) of NaH obtained from DFT is 22.93 GPa, while the DP model predicts a value of 23.97 GPa. Compared with the ReaxFF result of 29.2 GPa [12], the DP model shows much better agreement with the DFT-calculated value and is also closer to the experimental value of 19.4 ± 0.2 GPa [33]. This work also used DPMD and DFT to calculate the pressure derivative of the bulk modulus for NaH, obtaining consistent results. It is worth noting that the bulk modulus generally decreases with increasing temperature due to lattice expansion and anharmonic phonon effects, while the pressure derivative of the bulk modulus shows a relatively weak temperature dependence ($B_{0}^{\prime }$). The DPMD and DFT results are smaller than the experimental results at both $B_{0}$ and $B_{0}^{\prime }$. The equilibrium lattice constant ($a_{0}$) calculated by DFT is 4.78 Å, while DPMD and ReaxFF give a lattice constant of 4.78 Å and 4.77 Å, respectively. The experimental value of the lattice constant at 298 K is 4.91 Å. These results indicate that the DP model retains near-DFT accuracy for both structural and elastic properties while providing a more accurate description than the empirical ReaxFF potential.

To further investigate the predictive accuracy of DPMD in vibrational dynamics, DPMD and the phonon spectrum of NaH (*Fm3̅m*) were used to predict the phonon spectrum and compared with the DFT results. As illustrated in Figure 2c, the phonon frequencies of NaH calculated by DFT are primarily distributed within 0~10.0 THz and 15~30 THz. The DP model reproduces the overall phonon-frequency distribution obtained from DFT, although deviations of up to approximately 1 THz are observed in the 15–30 THz range.

### 3.2. Molecular Dynamics Simulation of Clusters

**Figure 3:**
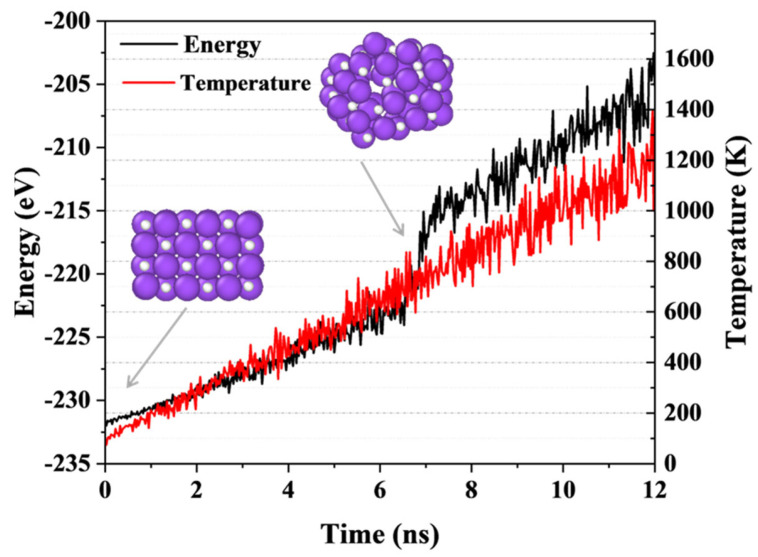
Potential energy and morphology changes of Na_48_H_48_ clusters during heating from 100 to 1200 K.

DPMD was used to investigate thermally induced H_2_ evolution in Na_x_Hγ clusters. Specifically, an NVT simulation was performed for a Na_48_H_48_ cluster heated from 100 to 1200 K. To do this, we performed a NVT simulation on a small NaH cluster Na_48_H_48_ by heating it from 100 to 1200 K. A velocity Verlet algorithm with a time step of 0.25 fs is used in all simulation runs. The heating rate was set at 9.17 × 10^−2^ K/ps (9.17 × 10^10^ K/s). The cluster is first minimized to find the nearest metastable state and then equilibrated at 100 K for 2 × 10^4^ steps. The temperature of the system is then increased linearly from 100 to 1200 K as:

(4) $$T\left(t\right)=T_{100\;K}+\lambda t$$

where λ is the heating rate. Figure 3 shows the time evolution of the potential energy PE of Na_48_H_48_ during the heating simulation from 100 to 1200 K. At 6.85 ns (700 K), the energy of Na_48_H_48_ increases markedly, which corresponds to the phase transition process; above this temperature, the cluster structure begins to become disordered. Nevertheless, no H_2_ formation is observed before the end of the simulation. This result differs from that reported by Ojwang et al. [12]. Using ReaxFF to examine the heating process of Na_48_H_48_, Ojwang et al. observed cluster decomposition at about 1100 K, whereas the phase transition behavior observed in this work at 700 K was not reported.

### 3.3. Molecular Dynamics Simulation of the Slab Models

**Figure 4:**
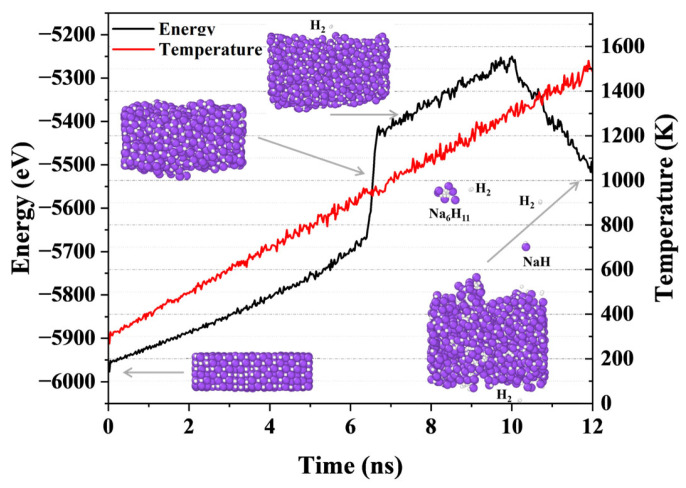
Potential energy and morphology changes of the NaH slab including 2400 Na and H atoms, during heating from 300 to 1500 K.

To investigate the thermal evolution of the NaH surface, DPMD simulations were performed using a six-layer NaH slab model. Generally, cluster structures are more sensitive to temperature changes; however, as no decomposition was observed in the aforementioned Na_48_H_48_ simulation, the slab was heated from 300 K to 1500 K, employing a heating rate of 0.1 K/ps (1.0 × 10^11^ K/s). The simulation results are shown in Figure 4. Consistent with Figure 3, phase transition was observed at 6.60 ns (951 K). This transition point temperature is higher than that observed for the cluster (700 K). At 10.00 ns (1318 K), the potential energy of the system decreases because the system releases stable H_2_ molecules, and the aggregation of Na leads to the rearrangement of the entire cluster into a more stable structure. At the end of the simulation, Na_6_H_11_, NaH and several H_2_ molecules were observed in the vacuum. This phenomenon did not occur in the cluster simulation, so the energy curve in Figure 3 does not show a decreasing trend. Notably, although large-scale decomposition of NaH occurs at 10.00 ns (1318 K), H_2_ release is already observed at 7.99 ns (1076 K). This temperature is close to the decomposition temperature (1031 K) of Na_48_H_48_ reported by Ojwang et al. [12].

**Figure 5:**
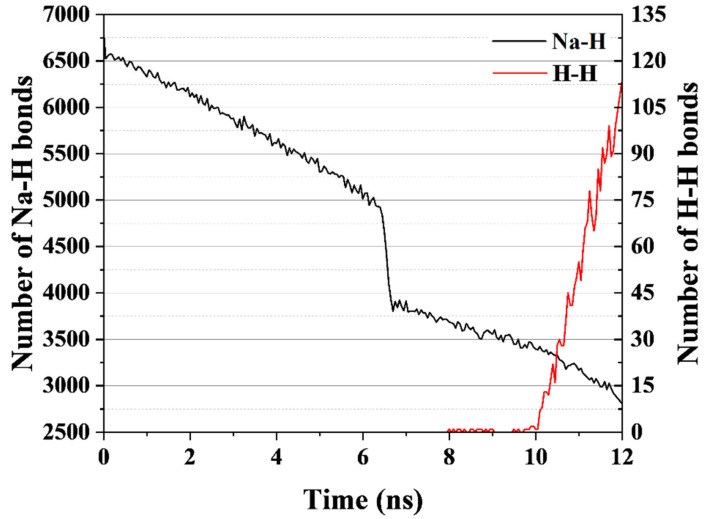
Changes in Na-H and H-H bonds in DPMD simulations of NaH slab during heating from 300 K to 1500 K.

To characterize the chemical rearrangements during NaH decomposition, the numbers of Na-H and H-H bonds were monitored along the DPMD trajectory. During the DPMD simulation of the slab model, trajectory frames were sampled every 50 ps to calculate the numbers of Na-H and H-H bonds, as shown in Figure 5. Because the atoms are in constant motion, the cutoff distances for bond lengths should be appropriately increased. Here, 1.2 times the Na-H bond length in the NaH crystal and 1.2 times the H-H bond length in molecular hydrogen are used as the cutoff distances. The Na-H cutoff distance is 2.39 × 1.2 = 2.87 Å, and the H-H cutoff distance is 0.75 × 1.2 = 0.90 Å. Since the initial model was a complete NaH slab model, the number of Na-H bonds was 6800. As the simulation temperature increased, the number of Na-H bonds decreased, with a significant decrease at 6.60 ns (951 K), corresponding to the rapid energy decrease stage in Figure 4, indicating that H atoms began to escape from the local NaH lattice environment. H-H bonds began to appear in the system between 7.99 ns (1076 K) and 10.00 ns (1318 K), corresponding to the generation of H_2_ in Figure 4. Subsequently, H_2_ molecules moved between the upper and lower surfaces of the model. After 10.00 ns (1318 K), the number of H-H bonds increased significantly, and the decrease in the number of Na-H bonds accelerated, corresponding to large-scale decomposition and H_2_ generation. This phenomenon was not observed in the simulations of the Na_48_H_48_ cluster; we attribute this to the relatively low temperature range used in the simulation. We subsequently performed a 5 ns DPMD simulation of the Na_48_H_48_ cluster in the 1000–1500 K range (Figure S2a) and observed a dissociation phenomenon. Analysis of the number of bonds further confirmed this (Figure S2b). To observe the H_2_ formation during the simulation process in more detail, we analyzed the distribution of the distance between adjacent H atoms over time, as shown in Figure S3. It can be seen that as the simulation progresses, the distance between adjacent hydrogen atoms is concentrated in the range of 0.72–0.80 Å, corresponding to the H-H bond length in hydrogen gas. These results suggest that the DP captures the main bond-rearrangement features associated with H_2_ formation and desorption in the simulated NaH systems.

To investigate the effect of high heating rates on apparent decomposition behavior, we performed additional isothermal simulations. A small NaH slab model (containing 150 Na and 150 H atoms) was equilibrated at two selected temperatures of 1250 K and 1300 K for 0.2 ns. At 1250 K, no H_2_ molecules were observed during the simulation. By contrast, one H_2_ molecule formed during the 1300 K isothermal simulation (Figure S4). These results indicate that H_2_ formation in the ideal NaH slab model is strongly temperature-dependent and kinetically limited on a nanosecond timescale. Therefore, the apparent decomposition temperature observed in continuously heated simulations is influenced by the rapid heating protocol and finite simulation time and should not be interpreted as the equilibrium decomposition temperature.

The present DPMD results can be qualitatively compared with the earlier ReaxFF simulations of NaH clusters reported by Ojwang et al. However, such a comparison should be regarded as qualitative rather than a direct one-to-one benchmark, because the predicted decomposition behavior can be strongly affected by the simulation protocol. In addition to the difference in the potential-energy model, factors such as the initial cluster configuration, heating rate, and total simulation time may all influence the apparent onset temperature and the observed structural pathway.

The thermal decomposition temperature of hydrogen storage materials is one of the most important parameters in practice. Grochala et al. [35] experimentally measured the thermal decomposition temperature of NaH to be 698 K, and Ke et al. [9] subsequently calculated it to be 726 K based on theoretical thermodynamics. However, the results of the DPMD simulations differ significantly from those reported in the literature, which may be due to several reasons. Generally, 698 K usually corresponds to thermal analysis, equilibrium or quasi-equilibrium judgments, while the actual dehydrogenation rate is also affected by sample size, defects, surface conditions, atmosphere, and heating rate. In the DPMD simulations, an ideal, clean, finite-size slab was used, where there are fewer defects, and the kinetics may actually be slower.

## 4. Discussion and Conclusions

This study developed a high-accuracy deep neural network potential for the NaH system using the DP-GEN active learning framework, achieving large-scale atomic-level simulations with near-DFT accuracy. Benchmark results show that the DP model accurately reproduces the energy, force, equation of state, elastic properties, and phonon spectrum obtained from DFT calculations, and significantly outperforms the empirical ReaxFF potential in describing the energy distribution and structural properties of NaH. Furthermore, the DP model agrees well with experimental data on key physical quantities such as lattice constant and bulk modulus. DPMD simulations of Na_48_H_48_ clusters indicate that the ordered solid-state atomic structure begins to break down at approximately 700 K, whereas in the slab model, this transition takes place at 951 K. At 1076 K, H-H bonds begin to form within the system, and H_2_ enters the vacuum layer. Significant decomposition is accompanied by Na-H bond cleavage and by H-H bond formation at 1318 K. However, it is important to note that the surface studies in this work only considered ideal, clean surfaces in a vacuum. In actual experiments, surface defects and environmental factors also influence NaH decomposition, which will be the next target for DPMD simulations. It should be emphasized that the heating rates used in the current DPMD simulations are much higher than those in experiments. Therefore, the transformation and decomposition onset temperatures obtained here should not be directly regarded as experimental decomposition temperatures. Instead, the primary significance of the simulations lies in revealing the qualitative sequence of events preceding hydrogen release from the NaH-containing cold-trap deposits. Specifically, prior to H_2_ release, the NaH undergoes lattice disruption, Na-H bond cleavage, and H-H bond formation. Overall, this study, by combining first-principles calculations and large-scale molecular dynamics simulations, establishes a reliable theoretical framework for studying the decomposition of NaH. The insights gained from this study deepen our understanding of hydrogen release mechanisms and provide valuable guidance for the safe handling and regeneration of sodium hydride cold trap systems in nuclear applications.

## Figures and Tables

**Table 1:** Simulation settings of the 9 DP-GEN iterations. The number of structures (N_s_) used for DPMD simulations ranges from 5 to 10, including the 5 cluster structures (Na_48_H_48_, Na_48_H_36_, Na_48_H_24_, Na_48_H_12_ and Na_48_) in the initial training. The length of DPMD trajectory (L_t_ in ps) is set from 0.1 ps to 1 ns. Four temperatures (T in K) ranging 100–1600 K are set for each iteration. All simulations were conducted at the pressure of 1 atm. The total number of atomic configurations (N_c_) that have been explored in each iteration.

Iter.	N_s_	T	Ensemble	L_t_	N_c_
1	5	100, 600, 1100, 1600	NVT	0.1	200
2	5	100, 600, 1100, 1600		0.5	1000
3	5	100, 600, 1100, 1600		1	2000
4	5	100, 600, 1100, 1600		5	10,000
5	10	100, 600, 1100, 1600	NVT	5	4000
6	10	100, 600, 1100, 1600		10	8000
7	10	100, 600, 1100, 1600		50	20,000
8	5	100, 600, 1100, 1600	NVT	100	2000
9	5	100, 600, 1100, 1600		1000	10,000

**Table 2:** The number of structures in the training set and the performance of the DP model on the subsets.

Structure		Number of Structures	Energy MAE (meV/atom)	Energy RMSE (meV/atom)	Force MAE (meV/Å)	Force RMSE (meV/Å)
Bulk	Na_32_	1500	6.20	7.43	10.60	14.75
	Na_32_H_32_	4000	5.01	5.98	12.09	15.90
Cluster	Na_48_	150	4.39	5.66	23.93	30.51
	Na_48_H_12_	537	3.84	4.88	38.20	49.56
	Na_48_H_24_	628	3.46	4.31	43.23	55.96
	Na_48_H_36_	647	3.39	4.43	45.85	59.45
	Na_48_H_48_	552	3.46	4.62	41.89	55.77
Gaseous	H_2_	400	6.58	8.61	34.12	41.30

**Table 3:** Equilibrium lattice constant $a_{0}$ (Å), bulk modulus $B_{0}$ (GPa) and the pressure derivative of the bulk modulus $B_{0}^{\prime }$ for NaH (*Fm3̅m*) calculated using DFT and ReaxFF. Experimental values from other workers are also shown.

	$a_{0}$	$B_{0}$	$B_{0}^{’}$	Temp
DFT (this work)	4.78	22.93	3.60	0
DPMD	4.78	23.97	3.59	0
ReaxFF [12]	4.77	29.20	-	0
Expt. [34]	4.91	19.04 ± 0.2	4.4 ± 0.5	298

## Data Availability

The original contributions presented in this study are included in the article/Supplementary Material. Further inquiries can be directed to the corresponding authors.

## Supplementary Materials

The following supporting information can be downloaded at: https://www.mdpi.com/article/10.3390/ma19153308/s1, Figure S1: (a) NaH unit cells used for testing KSPACING were displayed using VESTA 3.5.7 software; (b) The KSPACING test results showed that the maximum energy error measured by different KSPACING methods was 4.82 meV, which is within an acceptable error range. Figure S2: (a) Energy, temperature and (b) changes in the amount of Na-H and H-H variations in a DPMD simulation (time step 1 fs, heating rate 0.1 K/ps) of heating Na_48_H_48_ from 1000 K to 1500 K. Figure S3: Histograms showing the time distribution of distances between adjacent H atoms in the Na_48_H_48_ cluster during a temperature increase from 1000 K to 1500 K. Each histogram in the figure ranges from 0.04 Å. Figure S4: Snapshots of the small slab model (containing 150 Na and 150H) at the end of NVT simulations (simulation time 0.2 ns) at 1250 K and 1300 K, respectively. In both simulations, the atoms are in an amorphous structure. No H2 was produced in the 1250 K simulation, while H2 was produced in the 1300 K simulation, but the large-scale decomposition phenomenon after 10 ns as shown in Figure 4 did not occur.

## Abbreviations

The following abbreviations are used in this manuscript:

DP	Deep Neural Network Potential
DNN	Deep Neural Network
DFT	Density Functional Theory
MD	Molecular Dynamics
AIMD	ab initio Molecular Dynamics
VASP	Vienna Ab initio Simulation Package
GGA	Generalized Gradient Approximation
RMSE	Root Mean Square Error
MAE	Mean Absolute Error
