# Supplementary material for: Molecular Dynamics Study of Hydrogen Release from NaH Using Machine Learning Potential
Source: Materials (Basel). 2026 Aug 4;19(15):3308. doi: 10.3390/ma19153308 (PMC13467477; doi:10.3390/ma19153308)
Supplement: Supplementary file 1 [file materials-19-03308-s001.zip › materials-4352724-supplementary.pdf]

Molecular Dynamics Study of Hydrogen Release from NaH Using a Machine-Learning Potential

Ce Feng [1], Yuting Zhang [1], Xiao Zhang [1,2], Shikai Chang [3], Fuhao Zhang [1], Linyuan Cao [1,*], Jingya Dong [1,4,*] and Rongdong Wang [1,*]

1 Department of Reactor Engineering Technology, China Institute of Atomic Energy, Beijing 102413, China; fengce@cnncmail.cn (C.F.); zhangyuting@cnncmail.cn (Y.Z.); dgs_zhangxiao@cnncmail.cn (X.Z.); zhangfuhao@cnncmail.cn (F.Z.); caolinyuan@cnncmail.cn(L.C.); dongjingya@cnncmail.cn(J.D.);

2 School of Chemical Engineering and Technology, Tianjin University, Tianjin 300350, China

3 Jiangsu Key Laboratory for Carbon-Based Functional Materials & Devices, Institute of Functional Nano & Soft Materials (FUNSOM), Soochow University, Suzhou 215123, China; 20244014009@stu.suda.edu.cn

4 Institute of Nuclear and New Energy Technology, Tsinghua University, Beijing 100084, China

* Correspondence: caolinyuan@cnncmail.cn(L.C.); dongjingya@cnncmail.cn(J.D.); wangrongdong@cnncmail.cn(R.W.), Tel.: +86-10-69358162 (R.W.)

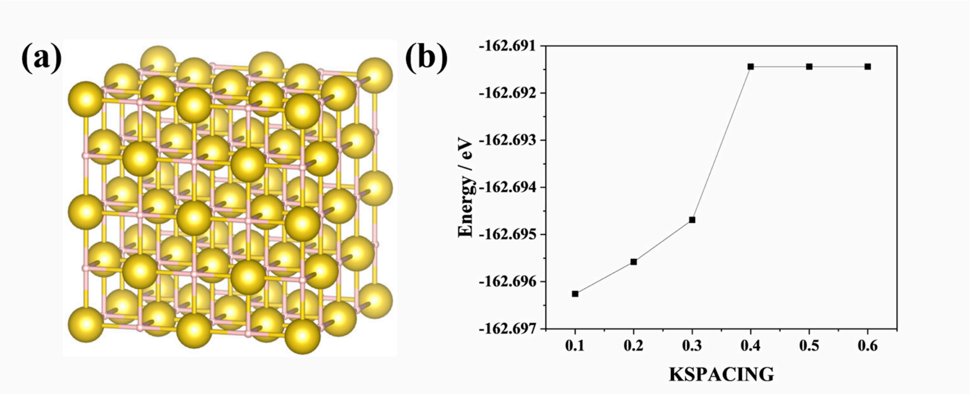

Figure S1. (a) NaH unit cells used for testing KSPACING were displayed using VESTA 3.5.7 software; (b) The KSPACING test results showed that the maximum energy error measured by different KSPACING methods was 4.82 meV, which is within an acceptable error range.

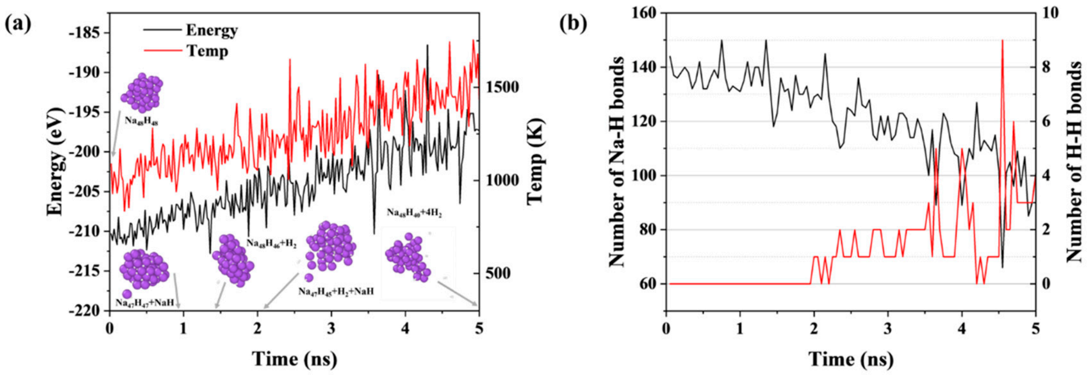

Figure S2. (a) Energy, temperature and (b) changes in the amount of Na-H and H-H variations in a DPMD simulation (time step 1 fs, heating rate 0.1 K/ps) of heating $Na_{48}H_{48}$ from 1000 K to 1500 K.

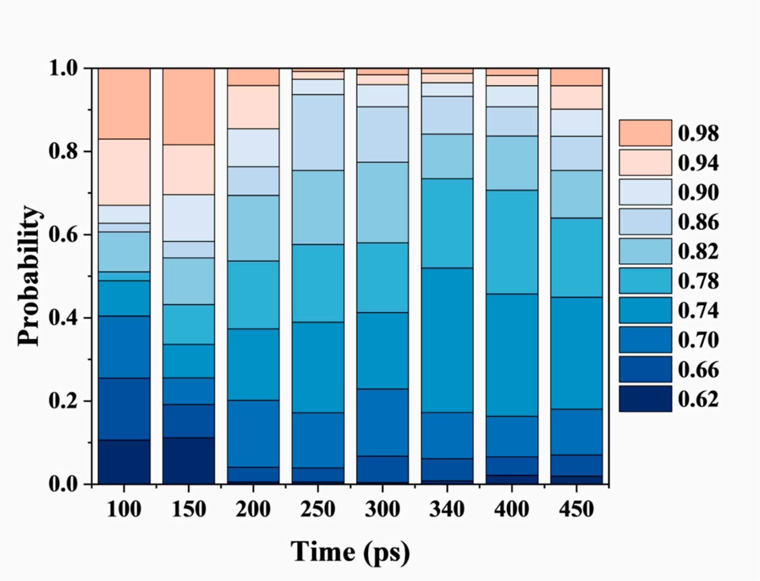

Figure S3. Histograms showing the time distribution of distances between adjacent H atoms in the $Na_{48}H_{48}$ cluster during a temperature increase from 1000 K to 1500 K. Each histogram in the figure ranges from 0.04 Å.

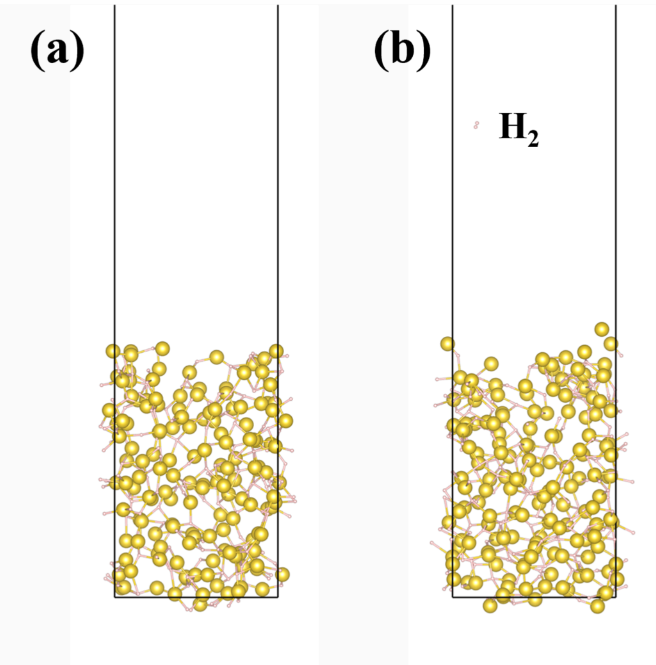

Figure S4. Snapshots of the small slab model (containing 150 Na and 150H) at the end of NVT simulations (simulation time 0.2 ns) at 1250 K and 1300 K, respectively. In both simulations, the atoms are in an amorphous structure. No H_2 was produced in the 1250 K simulation, while H_2 was produced in the 1300 K simulation, but the large-scale decomposition phenomenon after 10 ns as shown in Figure 4 did not occur.
